# Final 36-Month Outcomes from the Multicenter DynamX Study Evaluating a Novel Thin-Strut Novolimus-Eluting Coronary Bioadaptor System and Supporting Preclinical Data

**DOI:** 10.31083/j.rcm2408221

**Published:** 2023-08-01

**Authors:** Stefan Verheye, Mathias Vrolix, Matteo Montorfano, Francesco Giannini, Francesco Bedogni, Christophe Dubois, Bernard De Bruyne, Ricardo A. Costa, Daniel Chamié, José Ribamar Costa, Alexandre Abizaid, Antonio Colombo

**Affiliations:** ^1^Interventional Cardiology, ZNA Cardiovascular Center Middelheim, 2020 Antwerp, Belgium; ^2^Department of Cardiology, Ziekenhuis Oost-Limburg, 3600 Genk, Belgium; ^3^Interventional Cardiology Unit, San Raffaele Scientific Institute, 20132 Milan, Italy; ^4^Interventional Cardiology Unit, GVM Care & Research, Maria Cecilia Hospital, 48010 Cotignola (RA), Italy; ^5^Department of Cardiology, IRCCS Policlinico San Donato, 20097 San Donato Milanese-Milan, Italy; ^6^Department of Cardiovascular Medicine, Universitaire Ziekenhuizen Leuven, 3000 Leuven, Belgium; ^7^Cardiovascular Center, OLV Hospital, 9300 Aalst, Belgium; ^8^Cardiovascular Research Center, 04012-070 São Paulo, Brazil; ^9^Department of Biomedical Sciences, Humanitas University, 20090 Pieve Emanuele-Milan, Italy; ^10^Humanitas Clinical and Research Center IRCCS, 20089 Rozzano-Milan, Italy

**Keywords:** coronary artery disease, bioadaptor, drug-eluting stent, novolimus, target lesion failure, vessel motion, pulsatility, vasomotion, thrombosis

## Abstract

**Background:**

The DynamX Novolimus-Eluting Coronary Bioadaptor System (${\mathrm{DynamX}}^{®}$ Bioadaptor) has uncaging elements that disengage after the resorption of the polymer coating, aiming to restore vessel function in the treated segment and to avoid long-term adverse outcomes associated with the permanent caging of the coronary artery seen with conventional stenting.

**Methods:**

This prospective, multicenter, single-arm first-in-human study enrolled 50 patients in Belgium and Italy who were treated with the DynamX Bioadaptor. Eligible patients had *de novo* lesions in coronary arteries measuring between 2.5 and 3.5 mm in diameter and ≤24 mm in length. Clinical follow-up was performed up to 36 months. This analysis includes the intention-to-treat population and is based on data available. The preclinical studies include optical coherence tomography (OCT) analyses of 5 DynamX Bioadaptors implanted in 3 mini Yucatan pigs (at 3, 12 and 24 months), and assessment of smooth muscle cell gene expression profile in 8 pigs of which each was implanted with the DynamX Bioadaptor and the Xience drug-eluting stent. To assess the gene expression profile by quantitative real-time polymerase chain reaction, animals were sacrificed at 3, 6, 9 and 12 months.

**Results:**

Target lesion failure at 36 months was 8.7% (4/46), consisting of one clinically-driven target lesion revascularization and 3 cardiac deaths (all site-reported to be unrelated to the device or procedure). There were no additional target vessel revascularization and no definite or probable scaffold thrombosis. Preclinical data confirmed late lumen enlargement (from 7.02 ± 1.31 ${\mathrm{mm}}^{2}$ at baseline to 8.46 ± 1.31 ${\mathrm{mm}}^{2}$ at 24 months) and identified an increased expression of contractile genes around 9 months compared to a conventional drug-eluting stent.

**Conclusions:**

The DynamX Bioadaptor demonstrated very good 36-month clinical outcomes, highlighted by the absence of target-vessel myocardial infarction and definite or probable device thrombosis, and only one target lesion revascularization up to 36 months. These data are supported by preclinical studies that showed late lumen enlargement by OCT and an increased expression of contractile genes around 9 months compared to conventional drug-eluting stents, indicating faster vessel healing. Larger clinical studies are necessary to compare outcomes against contemporary drug-eluting stents.

**Clinical Trial Registration:**

https://clinicaltrials.gov/: NCT03429894.

## 1. Introduction 

While restenosis rates in percutaneous coronary interventions have been significantly reduced following the use of drug-eluting stents (DES), the permanent implant prevents normal vessel movement and function (expansion, contraction, rotation, vasomotion) due to permanent caging of the vessel [1].

Drug-coated balloons (DCBs) were developed to avoid a permanent implant, but they don’t support the scaffolding of the vessel, particularly to prevent early recoil, and come with a set of challenges such as drug delivery restricted to the time of inflation, and that only <10% of the coating is transferred to the vessel wall [2]. At present, DCBs are predominantly used to treat in-stent restenosis, but also in small vessels [3, 4]. Newer application modalities might be the combination of DCBs with DES to reduce the amount of implanted metal and the subsequent inhibition of vasomotion and the risk of long-term events [5, 6].

Fully bioresorbable scaffolds aim to provide scaffolding to the vessel during the initial healing phase and to be resorbed thereafter, ultimately allowing vascular restoration including lumen enlargement and restoration of vascular physiology and motion [1, 7, 8]. However, they have fallen out of favor through their intrinsic mechanical properties and as outcomes have not been comparable to contemporary DES, in particular in terms of higher restenosis and device thrombosis rates [2, 7], albeit optimized treatment strategies might improve outcomes [7].

The ${\mathrm{DynamX}}^{®}$ Bioadaptor (Elixir Medical Corporation, Milpitas, CA, USA) has been developed to overcome these challenges, with the unique feature that allows uncaging of the vessel after *in-vivo* degradation of the polymer base coat, which occurs over 6 months. The expansion segments also called “uncaging elements” are designed to disengage thus unlocking the artery to allow more normal vessel movement and function as compared to DES in the stented region [9, 10].

The DynamX Mechanistic study was initiated to evaluate the safety and performance of the DynamX Bioadaptor System in patients with *de novo* coronary artery lesions by assessing both clinical and imaging outcomes. Clinical and imaging outcomes out to two years have been published previously [9, 11], we herein report the final 36-month data.

In addition, two preclinical studies (porcine animal studies) were conducted to obtain further insights into the consequences of the uncaging of the bioadaptor. The first study assessed positive remodelling by optical coherence tomography (OCT) imaging. Another animal study assessed the levels of gene expression of the smooth muscle cells (SMC) in the device implanted neointimal tissue that is indicative of SMC phenotype switching during the arterial injury and subsequent healing upon peripheral coronary intervention [12, 13]. The results are included in this manuscript to provide insights into the biological response seen in the DynamX Mechanistic study.

## 2. Materials and Methods

### 2.1 Clinical Study

#### 2.1.1 Study Design

The study design has been described in detail previously [9]. The DynamX Mechanistic study is a prospective, multi-centre, non-randomized trial with consecutive enrollment. It was conducted at six sites in Belgium and Italy. Imaging follow-up with quantitative coronary angiography, intravascular ultrasound (IVUS) and OCT was performed at either 9 or 12 months and clinical follow-up was performed at 1, 6, 12, 24 and 36 months.

Eligible patients had single *de novo *coronary arteries measuring between 2.5 and 3.5 mm in diameter and ≤24 mm in length with a visually estimated stenosis of ≥50% and <90% and a thrombolysis in myocardial infarction (TIMI) flow of ≥2. Excluded were patients with acute myocardial infarction, left main disease, or in-stent restenosis. For further details, see ClincialTrials.gov NCT03429894. The study was approved by all ethics committees and all patients provided written informed consent.

Sources of bias were minimized through monitoring of electronic records (case report forms) with source document verification, imaging review through a core laboratory, and independent event adjudication by a clinical events committee. The study adhered to the Declaration of Helsinki, ISO14155:2011, and local regulations.

#### 2.1.2 Device and Procedure

**Fig. 1.**
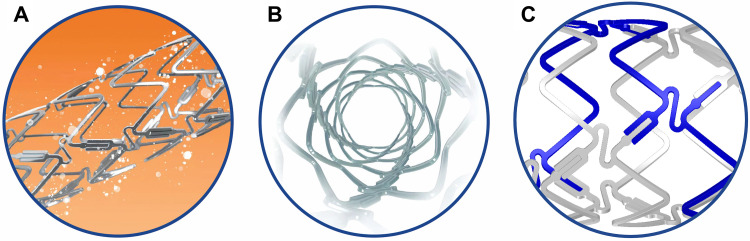
**DynamX Bioadaptor**. The drug elutes over 3 months (A). The polymer coating resorbs over 6 months and uncages the device circumferentially (B) at the U-shaped uncaging elements (C) while maintaining the longitudinal continuity of the three cobalt chromium strands in a helical pattern. Re-used with permission of Elixir Medical Corporation.

The DynamX Bioadaptor (Fig. 1) combines three 71 µm cobalt-chromium helical strands that are temporarily linked together by unique uncaging elements coated with a bioresorbable polymer. This creates a highly conformable scaffold with high acute compression resistance similar to DES. The thin polymer coating resorbs over six months allowing the cobalt-chromium helical strands to initiate the triple mechanism of unlocking the bioadaptor, uncaging the artery, and continue providing dynamic scaffolding after uncaging while uniquely adapting to vessel biomechanical forces and providing the necessary vessel reinforcement. The device has a crossing profile of ≤0.048” (≤0.050” for the 4.0 mm diameter). Maximum post-dilatation per instruction for use is 0.5 mm larger than the stent diameter.

The delivery system is comprised of standard materials. Two radiopaque balloon markers indicate the working length of the balloon and reflect the expanded bioadaptor length aiding to accurately position the bioadaptor and delivery system during implantation. The delivery system is designed to accommodate 0.014-inch or smaller diameter guide wires.

Implantation of the bioadaptor follows standard procedures for DES; dual antiplatelet therapy was recommended for at least 12 months.

#### 2.1.3 Endpoints and Definitions

The primary safety endpoint was target lesion failure (TLF, defined as a composite of cardiac death, target-vessel myocardial infarction, and clinically-driven target lesion revascularization) at 6 months, and the primary efficacy endpoint was change in the mean in-device area and lumen area at 9–12 months by IVUS.

Secondary endpoints at 3 years are TLF at other time points, target-vessel failure (TVF), defined as a composite of cardiac death, target-vessel myocardial infarction (TV-MI) and clinically-driven target vessel revascularization (CD-TVR); mortality, myocardial infarction (target-vessel and overall); clinically-driven target lesion revascularization (CD-TLR); overall TLR; CD-TVR; overall TVR; and definite or probable device thrombosis. Imaging endpoints have been reported previously [9, 11]. Myocardial infarction was defined as enzyme elevation of 2-times upper normal limit of creatinine kinase (CK) with an elevation of CK-MB; cardiac death, revascularization and device thrombosis were adjudicated according to Academic Research Consortium criteria [14].

#### 2.1.4 Statistical Analysis

The study was designed to confirm the performance and safety of the DynamX Bioadaptor and to generate hypotheses for future studies. Since there is no hypothesis testing, the sample size was not calculated based on the endpoint hypothesis. However, the sample size requirement was determined by assessing the minimal number of patients required to provide reliable and non-trivial results.

The analysis is based on the intention-to-treat principles. Qualitative data are presented as counts and percentages. Quantitative variables are presented as means and standard deviations. The results are based on the data available. Clinical data were analysed using ${\mathrm{Excel}}^{®}$ (Microsoft Corporation, Redmond, WA, USA) for Microsoft 365 MSO (Version 2109 Build 16.0.14430.20292).

### 2.2 Preclinical Studies

The study protocols of both studies were approved by the Institutional Animal Care and Use Committee (IACUC) and both studies were conducted in accordance with the Guide for the Care and Use of Laboratory Animals.

#### 2.2.1 OCT Analysis (Preclinical Study)

A total of 5 DynamX Bioadaptors (3.0 × 14 mm or 3.5 × 14 mm) were implanted in the coronary arteries of 3 Yucatan mini pigs for OCT imaging at multiple timepoints up to 24 months. Not all of the animals were imaged at all follow-up timepoints in order to avoid multiple procedures in the animals as per the IACUC requirements.

OCT imaging was performed at post-implant, and at 3, 12, and 24-month timepoints using the C7XR imaging system (LightLab, Westford, MA, USA). The OCT catheter was advanced distally to the implanted device and a motorized pullback was performed at a rate of 15 or 20 mm/sec to acquire images at a rate of 100 frames/sec. Images were acquired free of occlusion using a continuous flush of the contrast media. Three cross-sectional frames were chosen (proximal, mid and distal) to measure mean lumen diameter and mean device diameter to derive mean lumen area and mean device area at various time points. The analysis was performed with computer-assisted methods with the Image Pro Premier software (Version 9.2, Media Cybernetics, Rockville, ML, USA).

#### 2.2.2 Preclinical Assessment of Smooth Muscle Cell (SMC) Gene Expression Profile by Quantitative Real-Time Polymerase Chain Reaction (qRT-PCR)

A total of 8 pigs were implanted in the coronary arteries with 2 devices each of the DynamX Bioadaptor (test) and the Xience everolimus-eluting DES (control, Abbott Vascular, Santa Clara, CA, USA). Two pigs were euthanized at time points of 3, 6, 9, and 12 months post device implantation, the device implanted vessel segment was explanted, and the neointimal tissue harvested from the luminal side of the implanted DynamX Bioadaptor and the control Xience DES was isolated and stored in RNAlater solution. Total RNA purified from the neointimal tissue samples was reverse transcribed using First Strand cDNA Synthesis Kit (Syd Labs, Hopkinton, MA, USA) and quantified using qRT-PCR (Agilent MX3000P) at 3, 6, 9 and 12 months. **Supplementary Table 1** provides the target genes evaluated and the PCR primers employed in the qRT-PCR assay. The ΔCT (cycle threshold) method was used for relative quantification of gene expression in the test and control samples (each n = 4) from which the average fold change from the reference gene for the evaluated genes in the test and control samples was calculated. Reference gene beta-actin was used as normalization control [15].

## 3. Results

### 3.1 Clinical Study

#### 3.1.1 Baseline and Procedure

The 50 patients enrolled were 66.3 ± 8.9 years on average. 26% (n = 13) had diabetes, 70% (n = 35) had hypertension, 30% (n = 15) had prior myocardial infarction, and 38% (n = 19) prior percutaneous coronary interventions. Silent ischemia was present in 52%, stable angina in 22% and unstable angina in 4%. Asymptomatic post-myocardial infarction were 20%, and non-ST-elevation myocardial infarction 2%. Lesions were located in the left anterior descending in 44%, in the right coronary artery in 38%, and in the left circumflex artery in 18%. Moderate to severe calcification was present in 22% of lesions and 50% of lesions were type B2/C. Lesions were 11.1 ± 5.1 mm long with a mean reference vessel diameter of 2.91 ± 0.43 mm.

Pre-dilatation was performed in 96% of lesions and post-dilatation in 62%. One additional device was used to cover a dissection.

#### 3.1.2 Follow-Up

Four patients were lost to follow-up at 36 months and 3 patients died during the course of the study. All 3 patients had an uneventful procedure and index-hospital stay and all cardiac deaths were classified by the site as being not device- or procedure-related. The first death involved a 59-year-old male with multiple medical co-morbidities and Wernicke-Korsakoff syndrome. During the follow-up period, the patient had several hospital visits with only one cardiac-related visit on day 55 for atypical chest pain which spontaneously resolved. At day 255 he was found dead at home. The second death involved a 78-year-old male with hypertension and multiple comorbidities who was admitted to a non-study hospital for heart failure, where he died as a result of multi-organ failure on study day 267. Communication between the investigational site principal investigator and the cardiologist at the non-study hospital indicated that the patient did not experience any ischemic symptoms during the final hospitalization. The third cardiac death was reported on day 972 in a patient with a complex cardiac history not related to the index procedure (hospitalization for non-target vessel revascularization on post-procedure day 152, transcatheter aortic valve replacement on day 217, heart failure on day 553). On day 972, the patient died at home.

One clinically-driven TLR was reported in a patient on day 952 (Table 1). This patient underwent a protocol-required angiogram at 12 months which showed some narrowing of the target lesion (46% diameter stenosis and 0.91 fractional flow reserve, FFR) but did not warrant treatment at that time. On day 952, the patient was hospitalized for recurrent angina (nightly episodes) as well as rheumatic symptoms. Angiography showed severe in-stent restenosis (70% visual estimation, FFR 0.81) that was treated with a DES.

**Table 1.** **Clinical Outcomes at 36 months**.

	N = ${\mathrm{46}}^{1}$ patients
Target lesion failure	8.7% (4/46)
Cardiac death	6.5% (3/46)
Target-vessel myocardial infarction	0.0% (0/46)
Clinically-driven target lesion revascularization	2.2% (1/46)
Target vessel failure	8.7% (4/46)
${\mathrm{Clinically-driven target vessel revasclarization}}^{2}$	2.2% (1/46)
Device thrombosis, definite or probable	0.0% (0/46)

Data are displayed as % (n/N). ^1^4 patients lost-to-follow-up, ^2^includes the target lesion.

No non-clinically driven TLR or TVR occurred, but 5 non-TVR were reported (10.9%, on days 126, 152, 274, 535, and 1071).

### 3.2 Preclinical Studies

#### 3.2.1 OCT Analysis 

**Fig. 2.**
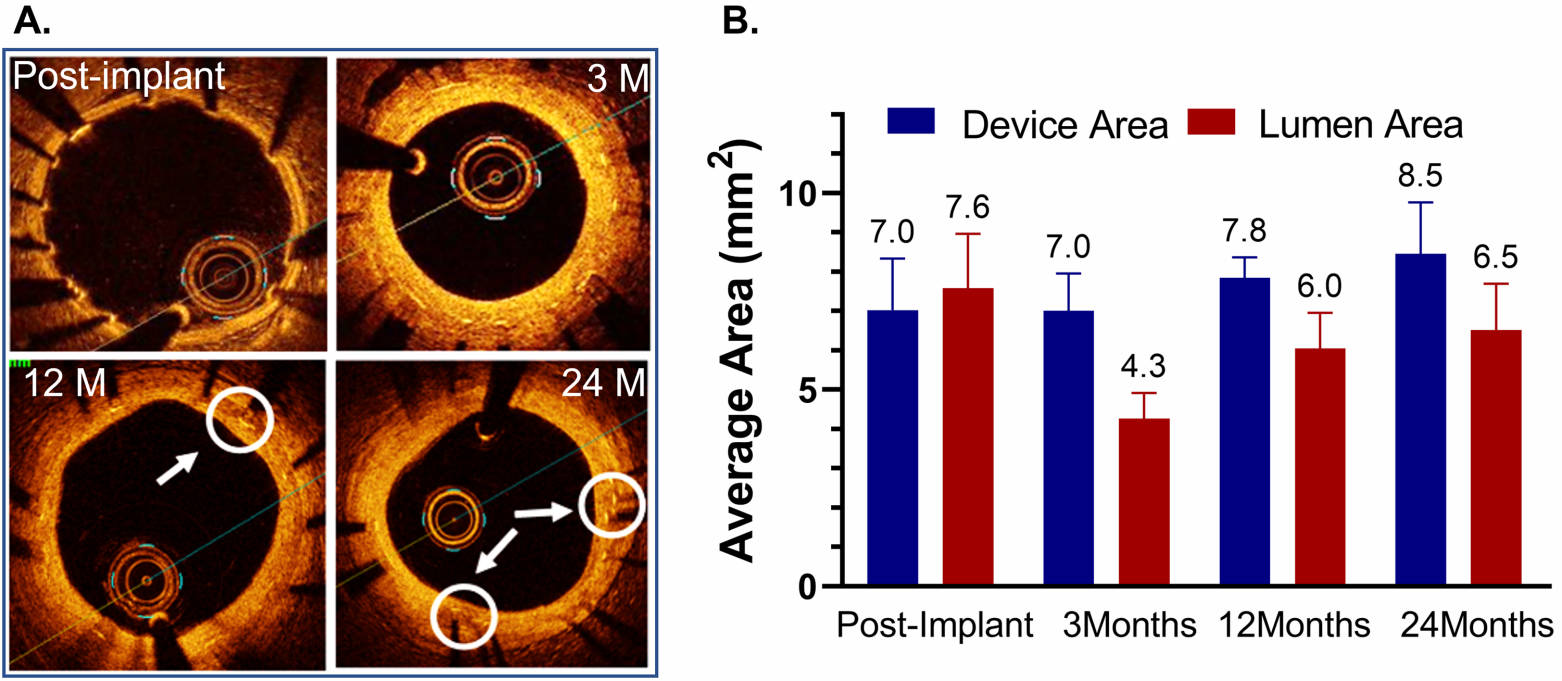
**Optical coherence tomography (OCT) after implantation of the DynamX Bioadaptor in Yucatan minipigs**. (A) Serial OCT images at various timepoint intervals show optimal strut apposition at post-implantation and uncaged elements at 12- and 24-month time points (circles & arrows). (B) The in-device area showed that there was only a marginal difference between post-implant and three months, indicating the absence of device recoil. The vessel lumen decreases at 3 months due to neointimal growth but eventually increases as the device area increases due to uncaging suggesting adaptive positive remodelling (n = 2–5). Used with permission of Elixir Medical Corporation.

OCT images of the test DynamX Bioadaptor were performed serially at multiple time points demonstrating good strut apposition to the vessel wall for the test and control devices (Fig. 2A). Since multiple follow-up imaging procedures were planned in the study, not all the animals were imaged at all follow-up time points in order to avoid multiple procedures in the animals as per the IACUC requirement. Hence an unpaired analysis of serial images was performed (Fig. 2B). Following uncaging (~6 months), imaging measurements showed that the mean device area had increased from 7.02 ± 1.31 ${\mathrm{mm}}^{2}$ at post implantation to 7.85 ± 0.52 ${\mathrm{mm}}^{2}$ at 12 months and 8.46 ± 1.31 ${\mathrm{mm}}^{2}$ at 24 months.

#### 3.2.2 Preclinical Smooth Muscle Cell Gene Expression Profile by qRT-PCR

**Fig. 3.**
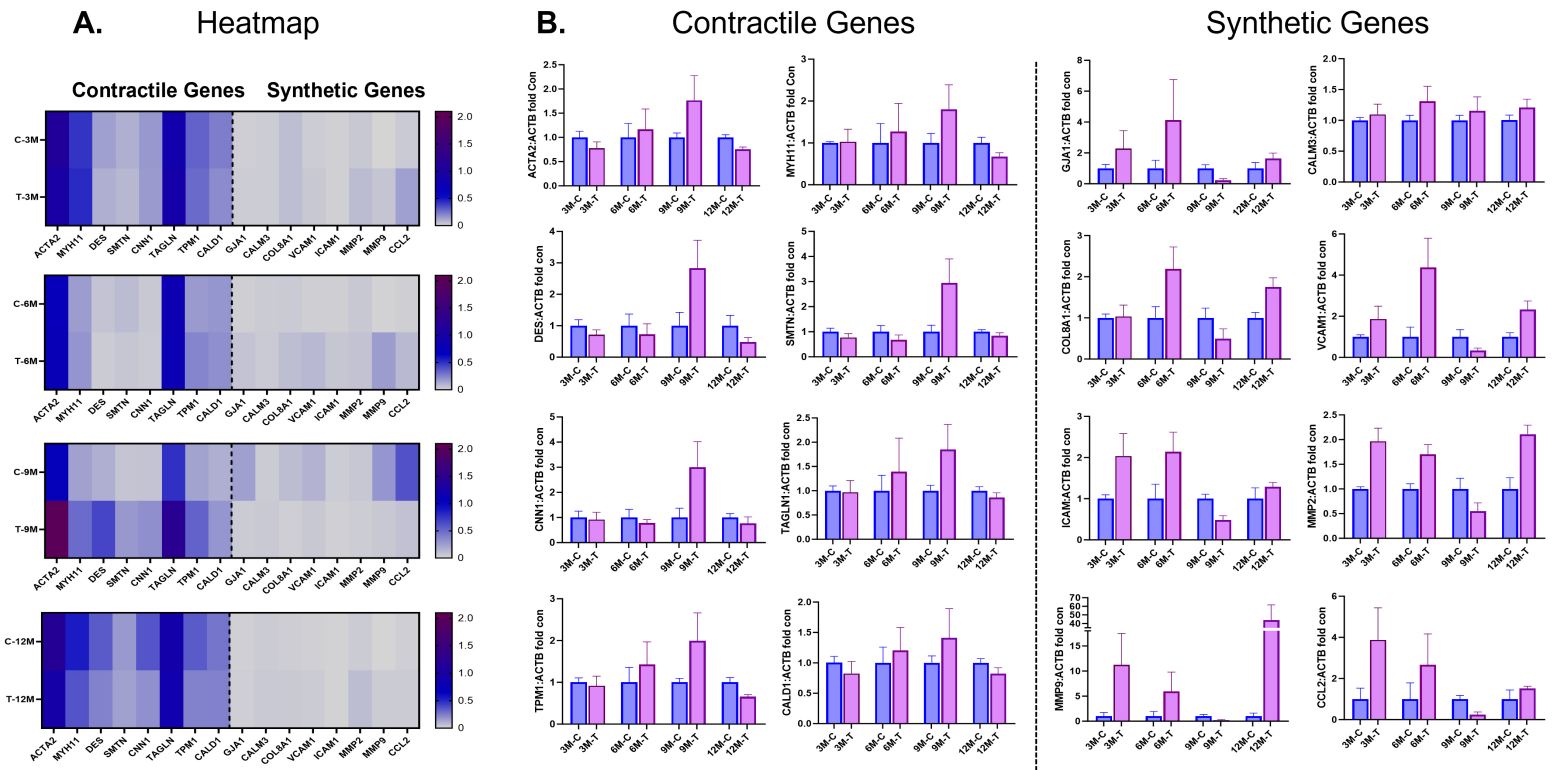
**Contractile and synthetic gene expressions at follow-up**. (A) Heatmap and (B) individual gene expression data showing comparison of smooth muscle cell (SMC) contractile and synthetic genes in neointimal samples between the test (DynamX Bioadaptor) and control (Xience stent) treatment. C-control, M-month, *T*-test. Used with permission of Elixir Medical Corporation. *ACTA2*, α smooth muscle cell actin; *CALD1*, heavy caldesmon; *CALM3*, calmodulin; *CCL2*, Monocyte chemoattractant protein 1; *CNN1*, smooth muscle calponin; *COL8A1*, collagen, type VIII, alpha 1; *DES*, desmin; *GJA*, connexin 43; *ICAM*, intercellular adhesion molecule; *MMP*, matrix metalloproteinase; *MYH11*, smooth muscle cell myosin heavy chain; *SMTN*, smoothelin; *TAGLN*, transgelin or smooth muscle 22 α; *TPM*, smooth muscle α tropomyosin; *VCAM*, vascular cell adhesion molecule; ACTB, actin beta.

As seen in the heatmap (Fig. 3), at early time points of 3 and 6 months, before and at the time of uncaging of the DynamX Bioadaptor, there is minimal difference in the expression of known synthetic and contractile genes in the neointimal tissue of vessels treated with either the DynamX Bioadaptor or the control Xience DES. At 9 months, 3 months after uncaging of the bioadaptor, the mRNA expression of well-known differented SMCs marker genes, namely, α smooth muscle actin (*ACTA2*), smooth muscle myosin heavy chain (*MYH11*), Desmin (*DES*), Smoothelin (*SMTN*), smooth muscle Calponin (*CNN1*), Transgelin or smooth muscle 22α (*TAGLN*), and smooth muscle α-tropomyosin (*TPM*), were 1.5–3 fold up regulated in the neointima of DynamX treated vessels versus controls, whereas dedifferentiated, proliferative and synthetic SMC gene markers such as collagen VIII (*COL8A*), matrix metalloproteinase (*MMPs*), intercellular adhesion molecule (*ICAM*), vascular cell adhesion molecule (*VCAM*), and heavy Caldesmon (*CALD1*) were simultaneously downregulated. Over a longer period at 12 months, the contractile gene expression of the control Xience-DES became comparable to the DynamX Bioadaptor.

## 4. Discussion

These are the first 36-month outcomes presented for the novel DynamX Bioadaptor device platform. Only one TLR occurred and no definite or probable device thrombosis were reported.

The DynamX Bioadaptor shares some advanced features with contemporary second-generation DES, i.e., it consists of ultrathin (71 µm) cobalt-chromium struts covered with a biodegradable polymer that elutes novolimus [9]. Its innovative design, however, includes uncaging elements within the bioadaptor pattern that unlock the implant and permit the improvement in vessel movement and function after *in-vivo* resorption of the bioresorbable polymer coating.

Thus, the DynamX Bioadaptor is the first permanent coronary artery implant designed to improve vessel function and physiology. The ability to improve the vessel function through disengagement of the uncaging elements was demonstrated at 9- to 12-month imaging follow-up [9, 10, 11] through:

(a) Positive arterial remodelling. Unlike DES, where the lumen is expected to decrease over time, the late vessel and bioadaptor expansion, measured by IVUS, compensates for the increase in neointima that occurs with DES implantation and allows for the maintenance of the lumen area that was achieved with the initial deployment (increase in in-device area from 7.39 ± 1.20 ${\mathrm{mm}}^{2}$ at baseline to 7.74 ± 1.46 ${\mathrm{mm}}^{2}$ at 9- to 12-month follow-up, *p* = 0.0005, increase in vessel area from 14.11 ± 2.99 ${\mathrm{mm}}^{2}$ to 14.54 ± 3.12 ${\mathrm{mm}}^{2}$, *p* = 0.02, and maintained lumen area with 7.39 ± 1.20 ${\mathrm{mm}}^{2}$ at baseline and 7.36 ± 1.31 ${\mathrm{mm}}^{2}$ at follow-up, *p* = 0.59).

(b) Improved vessel pulsatility. The vessel pulsatility in the treated segment increased in response to the cardiac cycle (46% improvement in maximum lumen area change at follow-up by IVUS), resulting in a reduction of the segmental mismatch in area compliance between the treated and untreated adjacent segments.

(c) Improved vasomotion. Vasomotion in response to nitroglycerine increased from 0.03 ${\mathrm{mm}}^{2}$ post-procedure to 0.17 ${\mathrm{mm}}^{2}$ at follow-up.

(d) Restoring angulation. There was a return towards baseline angulation at follow-up (from a mean of 137.6 ± 16.2° at baseline, over 157.5 ± 14.5° post-procedure to 149.7 ± 16.1° at 9- to 12-month follow-up, measured by quantitative coronary angiography).

(e) Reduced stress. The peak stress within the bioadaptor was reduced by 70% after uncaging, evaluated by finite element analysis.

Likewise, in a preclinical animal study with serial OCT assessment at multiple timepoints, the mean device area did only marginally change from post-procedure to 3 months, indicating the absence of acute or subacute vessel recoil. But after uncaging, at 12 and 24 months, the mean device area increased, further demonstrating the ability for positive adaptive remodelling that compensates for the neointimal growth, as seen for the lumen diameter that decreased at three months, but increased again after uncaging.

Another preclinical study showed that the neointimal covering is majorly constituted of SMCs that are not terminally differentiated and may modulate their phenotype in response to local microenvironmental changes such as vascular injury [12, 13]. An initial proliferative and dedifferentiated state of neointimal SMCs is characterized by upregulation of synthetic or activation marker genes, which eventually reverses into a low proliferative and differentiated state characterized by upregulation of contractile genes, e.g., ACTA2, DES, smooth muscle myosin heavy chain (SM-MHC), smooth muscle protein 22-α (SM22α), etc. as the healing progresses to finally regain the contractile and physiological properties [16, 17, 18]. Increased expression of contractile genes in the neointima explanted from DynamX vs Xience DES-treated vessel segment at 9 months indicates faster vascular healing with a bioadaptor after uncaging compared to a caged DES [13]. This improvement in vessel function might have contributed to the good outcomes of the DynamX Mechanistic study.

Acknowledging the limitations of the small patient population enrolled in the DynamX Mechanistic study, outcomes are at least comparable to commercially available second-generation DES systems. Twelve-month data have been presented previously and reported low in-device late lumen loss (0.12 ± 0.18 mm by quantitative coronary angiography analysis), low %volume obstruction (3.39 ± 4.66% by IVUS analysis) and nearly complete neointimal coverage (98.95 ± 2.85% covered struts by OCT) [9]. The OCT data also showed sufficient neointimal growth to cover the bioadaptor struts with a thickness that is in the expected range for commercially available DES [19, 20].

At 36 months, only one CD-TLR and no additional CD-TVR occurred. The absence of any non-target lesion CD-TVR is possibly associated with the improvement in segmental compliance mismatch, reducing the irritation at the stent edges. Certainly, the CD-TLR rate of 2.2% at 36 months compares well to other contemporary DES. In the BIONYX trial, the 36-month CD-TLR rate was 4.7% for the zotarolimus-eluting Resolute Onyx stent (Medtronic, Santa Rosa, CA, USA) and 4.6% for the Orsiro sirolimus-eluting DES (Biotronik AG, Buelach, Switzerland) [21]; in the BIOFLOW-V trial, it was 3.2% for Orsiro versus 6.7% for the everolimus-eluting Xience DES (Abbott Vascular, Santa Clara, California) [22]; in the BIO-RESORT trial the 36-month CD-TLR rate ranged from 2.9% to 3.8% for the Orsiro DES, the Resolute Integrity zotarolimus-eluting stent, and the Synergy everolimus-eluting stent (Boston Scientific, Marlborough, Massachusetts) [23]; and in the TALENT trial, it was 5.0% for the Supraflex sirolimus-eluting stent (Sahajanand Medical Technologies, Surat, India) and 5.9% for Xience [24].

Noteworthy, no thrombotic events occurred (0.0% target-vessel myocardial infarction and 0.0% definite or probable device thrombosis), as compared to definite or probable stent thrombosis rates that range from 0.5% to 1.4% in the BIONYX, BIO-RESORT and TALENT trials [21, 23, 24].

### Limitations

Our study has several limitations: It included a limited number of subjects and a selected patient population with relatively simple lesions; the outcomes need to be confirmed in larger trials with more complex patients. Furthermore, the single-arm design precludes a direct comparison to other DES. Nevertheless, these first 36-month outcomes of this innovative device are relevant and the trial was listed in the Advances in Clinical Cardiology Summary of Key Clinical Trials [25]. Several trials are currently ongoing to assess the device in larger patient populations with more complex lesions, and to compare it against contemporary DES in randomized settings. The animal studies have the inherent limitation that the vessels are not atherosclerotic.

## 5. Conclusions

In conclusion, the DynamX Bioadaptor demonstrated very good safety and performance outcomes. The 36-month TLF-rate was low with the absence of any target-vessel myocardial infarction, and only one target lesion revascularization. No definite or probable device thrombosis was reported. These outcomes are potentially related to an improved vessel function in the treated segment, as demonstrated through intracoronary imaging and as confirmed in preclinical studies with late lumen enlargement assessed by OCT and an increased expression of contractile genes around 9 months compared to a conventional DES which is indicative for vessel healing. Larger, randomized studies are necessary to corroborate these findings and to compare long-term outcomes against contemporary DES.

## Data Availability

Data are available from the corresponding author upon reasonable request, but with an embargo of 12 months.

## Abbreviations

CD, clinically-driven; DES, drug-eluting stent; IVUS, intravascular ultrasound; OCT, optical coherence tomography; SMC, smooth muscle cells; TLF, target lesion failure; TLR, target lesion revascularization; TVF, target vessel failure; TV-MI, target-vessel myocardial infarction; TVR, target vessel revascularization.

## Supplementary Material

Supplementary material associated with this article can be found, in the online version, at https://doi.org/10.31083/j.rcm2408221.
