# Supplementary material for: Final 36-Month Outcomes from the Multicenter DynamX Study Evaluating a Novel Thin-Strut Novolimus-Eluting Coronary Bioadaptor System and Supporting Preclinical Data
Source: Rev Cardiovasc Med. 2023 Aug 1;24(8):221. doi: 10.31083/j.rcm2408221 (PMC11266769; doi:10.31083/j.rcm2408221)
Supplement: Supplementary file 1 [file 2153-8174-24-8-221-s1.docx]

*Original Research*

Final 36-month outcomes from the multicenter DynamX Study evaluating a novel thin-strut Novolimus-Eluting Coronary Bioadaptor System and supporting preclinical data

Supplementary Table 1. Real-time PCR primers.

| **Gene Symbol** | **Description** | **Forward Primer (5´→3´)** | **Reverse Primer (5´→3´)** |
| --- | --- | --- | --- |
| **Contractile Phenotype Associated Genes** | | | |
| ACTA2 | α smooth muscle actin (ACTA-2) | GGTTCTGGGCTCTGTAAGGC | GTCCCACAATGGACGGGAAA |
| MYH11 | Smooth muscle myosin heavy chain (MYH11) | GGTTACATCGTGGGAGCCAA | GCCCTCCAAGAGCAAGTCAT |
| DES | Desmin | GCCGGATCAACCTCCCTATC | ACCTCAGAACCCCTTTGCTC |
| SMTN | Smoothelin | GTGATGGGATGGCCTTCTGT | CAAAGTTCTGGCGCCGATTC |
| CNN1 | Smooth muscle Calponin | GTCGGCTGAGGTCAAGAACA | CCTCGATCCACTCTCGAAGC |
| TAGLN | Transgelin or smooth muscle 22α (TAGLN) | TCCAGGTCTGGCTGAAGAATG | GTTTGGAGCCGTCAGGATACA |
| TPM1 | Smooth muscle α-tropomyosin | ATGCCGACCGCAAGTATGAA | CCTTCTGAGAGTTCAGCCCG |
| CALD1 | Heavy Caldesmon (H-Caldesmon) | TGCGTACCCGCATTATCTGG | TCCTCTGGTAGGCGATCCTT |
| **Synthetic Phenotype Associated Genes** | | | |
| GJA1 | Connexin 43 (Cx43) | GTTTCCTCTCTCGTCCCACG | TGTTCAAGGCGAGAGACACC |
| CALM3 | Calmodulin | GCCTCTGGGACAAGGAAGTC | GGGTGGAGGGCAGATGAAAT |
| COL8A1 | Collagen VIII | CAAGGAACTGCCACACATGC | TTCTTTGCCTTTCTTGGGTGC |
| VCAM1 | Vascular cell adhesion molecule-1 (VCAM-1) | GCGAGTCCTCCCTGTCTTTC | CGTGGATCTGGTCCCGTTAG |
| ICAM1 | Intercellular adhesion molecule-1 (ICAM-1) | CACACCTTGCTACCCCTGAG | GCTGGGAACAGTCCATCCAA |
| MMP2 | Matrix metalloproteinases-2 (MMP-2) | CCGACGTGGCCAATTACAAC | GGTCCAGATCAGGCGTGTAG |
| MMP9 | Matrix metalloproteinases-9 (MMP-9) | CATTCAAGGAGACGCCCACT | GCCTTTTGCGTTTCCGAAGT |
| CCL2 | Monocyte chemoattractant protein 1 (MCP-1) | AAACGGAGACTTGGGCACAT | GCAAGGACCCTTCCGTCATC |
| **House-keeping Gene** | | | |
| ACTB | actin beta | ACGATATTGCTGCGCTCGT | AGCATCGTCGCCCGCAAAG |

PCR-polymerase chain reaction.
